# Borderline Personality Disorder: Why ‘fast and furious’?

**DOI:** 10.1093/emph/eow002

**Published:** 2016-02-28

**Authors:** Martin Brüne

**Affiliations:** LWL University Hospital, Department of Psychiatry, Psychotherapy and Psychiatric Preventive Medicine, Division of Cognitive Neuropsychiatry and Psychiatric Preventive Medicine, Ruhr-University Bochum, Germany

**Keywords:** Borderline Personality Disorder, Life History Theory, adversity, interpersonal opportunism, psychotherapy, deficit model

## Abstract

The term ‘Borderline Personality Disorder’ (BPD) refers to a psychiatric syndrome that is characterized by emotion dysregulation, impulsivity, risk-taking behavior, irritability, feelings of emptiness, self-injury and fear of abandonment, as well as unstable interpersonal relationships. BPD is not only common in psychiatric populations but also more prevalent in the general community than previously thought, and thus represents an important public health issue. In contrast to most psychiatric disorders, some symptoms associated with BPD may improve over time, even without therapy, though impaired social functioning and interpersonal disturbances in close relationships often persist. Another counterintuitive and insufficiently resolved question is why depressive symptoms and risk-taking behaviors can occur simultaneously in the same individual. Moreover, there is an ongoing debate about the nosological position of BPD, which impacts on research regarding sex differences in clinical presentation and patterns of comorbidity.

In this review, it is argued that many features of BPD may be conceptualized within an evolutionary framework, namely behavioral ecology. According to Life History Theory, BPD reflects a pathological extreme or distortion of a behavioral ‘strategy’ which unconsciously aims at immediate exploitation of resources, both interpersonal and material, based on predictions shaped by early developmental experiences. Such a view is consistent with standard medical conceptualizations of BPD, but goes beyond classic ‘deficit’-oriented models, which may have profound implications for therapeutic approaches.

## INTRODUCTION

The term Borderline Personality Disorder (BPD) refers to a psychiatric condition that is characterized by unstable interpersonal relationships, fear of abandonment, difficulties in emotion regulation, feelings of emptiness, chronic dysphoria or depression, as well as impulsivity and heightened risk-taking behaviors. Paranoid ideation and dissociative states are also transient features of the syndrome (Table 1). Moreover, many patients with BPD show recurring self-injurious or suicidal behavior [1]. BPD has a lifetime prevalence of about 6%. It is much more common in clinical settings, thus rendering BPD highly relevant for health care providers and public health in general [2].

**Table 1.** Descriptive diagnostic criteria of Borderline Personality Disorder according to the DSM-5

Fear of abandonment
Unstable and intensive relationships with rapid changes between idealization and derogation
Identity disorder
Impulsivity (spending money, sexuality, substance abuse, other risk-taking behaviors)
Recurrent suicidal behavior, threat of committing suicide or self-injurious behavior
Emotional instability
Feelings of emptiness
Inappropriate anger, uncontrolled aggression
Stress-dependent paranoid ideation or dissociative symptoms

A diagnosis is based on the presence of at least five of the following signs or symptoms

Etiological models of BPD suggest that the development of ‘mistrustful inner working models’ based on insecure attachment predisposes to perceiving others as untrustworthy and rejecting [3–5]. Causal factors in this development include childhood trauma such as emotional neglect or physical and sexual abuse, though associating BPD with traumatic events alone is an oversimplification [6–8]. The contribution of genetics to BPD is inconclusive, but heritability of BPD seems to be significant [9, 10]. Taken together, the experience of early adversity, particularly the emotional unresponsiveness of attachment figures, trauma or abuse, coins an individual’s expectations with regard to future resource availability, including the quality of interpersonal relationships in terms of others’ reliability and trustworthiness [5].

BPD is often a comorbid condition of other psychiatric disorders (formerly conceptualized as axis-I disorders according to DSM-IV), foremost depression, other personality disorders, and there seems to be syndromal overlap and/or comorbidity with bipolar disorder (BD), attention deficit/hyperactivity disorder (ADHD) and posttraumatic stress disorder (PTSD) [2, 11–14].

In keeping with traditional medical conceptualizations, many scholars see BPD as a clinical syndrome with identifiable brain lesions or defects, mainly affecting fronto-limbic connections, which account for patients’ emotional dysregulation, impulsivity and inability to cope with interpersonal distress [e.g. 15]. Such views are incompatible, however, with observations suggesting that interpersonal difficulties of individuals with BPD are largely absent outside emotionally challenging situations, and that over time many patients experience a substantial reduction in self-mutilating behavior and impulsivity, though full recovery is rare and interpersonal difficulties and emotional instability are more pervasive [16]. In fact, most psychiatric conditions worsen with increasing age, so, why should BPD be an exception? Another counter-intuitive issue pertaining to BPD is that risk-taking behavior and depression co-occur in the same condition, whereby people with depression are usually risk-averse, rather than risk-prone, the latter being a typical feature of BPD [17]. Finally, there is controversy about sex differences in prevalence and clinical presentation of BPD, much of which remains unresolved, possibly due to conceptual diversity [18–20].

In consideration of these conceptual inconsistencies, the present article seeks to shed a different light on BPD. It is proposed that some features of BPD can be better understood in a frame of reference taking into account insights from behavioral ecology. Accordingly, cognition, emotions and behaviors typical of BPD may become meaningful and comprehensive, sometimes even logical, when imagining a world that is dangerous and unpredictable, where a ‘fast and furious’ lifestyle may appear appropriate. Such a view does not contend that BPD is adaptive *per se*. Instead, it is suggested that individual signs and symptoms associated with BPD can be meaningfully integrated in a life history perspective, and that sub-threshold or ‘diluted’ phenotypes of BPD may well pay off reproductively (i.e. being adaptive in the biological sense), though perhaps at the expense of well-being and mental health. With regard to clinical implications, it is claimed that a behavioral ecological perspective may also shift focus in relation to psychotherapeutic goals away from fighting signs and symptoms (i.e. ‘dis-ease’) to views that aim at reframing an individual’s life history strategy in more functional ways by means of improving patients’ insight into and acceptance of the inter-relatedness of early life experiences with the pursuit of current bio-social goals.

## BEHAVIORAL ECOLOGY

Behavioral Ecology focuses on the variation in behavior between as well as within species and its contingency on environmental conditions. An important behavioral ecological concept, termed Life History Theory (LHT), concerns an organism’s differential allocation of resources to physical growth and reproduction. Put another way, there is a trade-off between an organism’s capacity to invest energy in somatic growth, as opposed to investment of energy in reproductive activity, resulting in different life history strategies (LHS) shaped by natural selection. Accordingly, growth rate, age and body size at sexual maturation, number and size of offspring, mortality rate, longevity, etc. are biological traits modeled by environmental contingencies [21].

The concept of LHT was originally applied to differences between species, with growing evidence for within-species differences in LHS [22]. That is, ecological (environmental) conditions (interacting with genetic factors) determine whether an individual adopts a ‘faster’ or ‘slower’ LHS, whereby current and future resource availability is estimated by observable cues or predicted based on prior experience acquired in early developmental stages [23]. Critical aspects involved in ‘decisions’ over faster or slower LHS concern the timing of biological maturation, current versus future reproduction, quality versus quantity of offspring, and quality versus quantity of parental care in offspring and mating [24, 25].

A wealth of research has shown that the principles of LHT apply to humans in the same way as to any other organism [24]. It is necessary to point out, however, that terms such as ‘strategy’ or ‘decision-making’ do not imply conscious reflection or intentional action. The timing of biological maturation, sexual activity and intensity of care for offspring is regulated by sex hormones, the stress response system and neuropeptides [26–28]. In a more general vein, however, LHS have also profound ramifications for the shaping of interpersonal behavior including cooperation, reciprocity, aggression and pair-bonding, as well as for neurocognitive domains, such as risk-taking, executive functioning and inhibitory control [25]. According to the ‘Adaptive Calibration Model’ individual differences in stress-regulation, as a function of complex gene–environment interaction, may translate into different adaptive strategies, which may shift one’s somatic development and psychological mechanisms toward a ‘faster’ or ‘slower’ LHS [25, 27].

In support of theories about LHS, abundant research suggests that differences in early environmental conditions shape an individual’s LHS in predictable ways [29]. Central to this is the observation that the quality of parenting profoundly influences the way children develop ‘inner working models’ which in turn serve as a guideline for predicting future resource availability [3]. That is, children who grow up in an emotionally safe and stable familial environment learn to see the world as a safe place, in which stable relationships with trustworthy others (family, peers, partners) indicate the availability of social and material resources in the future. Accordingly, from the perspective of attachment theory, securely attached individuals tend to pursue slower LHS, that is, they tend to mature later, delay reproduction, are generally risk averse, and form stable long-term intimate relationships with partners. Such individuals are also cooperative, empathetic, display low levels of interpersonal aggression, and have good inhibitory control over impulses. In terms of personality traits, they score high on conscientiousness and agreeableness. In contrast, children who are exposed to environmental cues such as harsh parenting, violence or other sources of danger are more likely to develop an inner working model suggesting that future resource availability is unpredictable, thereby shifting LHS toward faster development, including earlier biological maturation, sexual activity and earlier reproduction [24, 29]. A faster LHS is more often associated with insecure attachment patterns, increased delay discounting, greater impulsivity, larger numbers of sexual partners, lack of reciprocity, reduced inhibitory control, increased risk-taking behavior and less parental effort. Moreover, several personality traits may also be linked to differential LHS, whereby conscientiousness and agreeableness may be the most relevant in this regard, whereas others such as extraversion, openness and neuroticism may be more ambiguous indicators of a particular LHS (Table 2) [30, 31].

**Table 2.** Prediction from LHT with regard to cognitive, emotional development, interpersonal behavior and physiology (modified after Del Giudice [25])

	‘Fast’ LHS	‘Slow’ LHS
Cognition/emotions	Low empathy^a^ ^[mixed evidence, 40]^	High empathy
	Heightened threat sensitivity^a^ ^[^42]	Low threat sensitivity
Neuropsychology	Low tolerance of frustration^a^ ^[^45^]^	High tolerance of frustration
	Poor executive control^a^ ^[^45^]^	Good executive control
Personality	Neuroticism ↑^a^ ^[^48^]^	Neuroticism ↓
	Agreeableness ↓^a^ ^[^50^]^	Agreeableness ↑
	Conscientiousness ↓^a^ ^[^49^]^	Coscnientiousness ↑
Temperament and character	Novelty seeking ↑^a^ ^[^48^]^	Novelty seeking ↓
	Harm avoidance ↓^a^ ^[^55–60^]^	Harm avoidance ↑^a^ ^[^48^]^
	Impulsivity ↑^a^ ^[^56, 74^]^	Impulsivity ↓
	Risk proneness ↑^a^ ^[^58, 59^]^	Risk proneness ↓
Interpersonal behavior	Opportunistic^a^ ^[^44, 49^]^	Altruistic
	Low parenting effort^a^ ^[^68^]^	High parenting effort
	Unstable intimate relationships^a^	Stable intimate relationships
Stress physiology	High cortisol^a^ ^[^35^]^	Low cortisol
	Reduced HRV	High HRV
Other biological markers	Early sexual maturation^a^ ^[indir. evidence, 64^^–^^66]^	Late sexual maturation
	High ‘allostatic load’	Low ‘allostatic load’

a Supportive evidence for Borderline Personality Disorder as a ‘fast’ Life History Strategy (LHS).

In line with LHT models of socialization, and consistent with the Adaptive Calibration Model, the experience of early adversity, particularly emotional unresponsiveness of attachment figures, trauma, abuse, coins an individual’s expectations with regard to future resource availability in terms of interpersonal relationships, i.e. trustworthiness, reciprocity and empathetic concern, suggesting that individuals would tend to maximize short-term benefits from interpersonal relationships, that is pursue a fast LHS [27, 29, 32].

Accordingly, the idea that BPD is typical of a ‘fast’ LHS has face value, because several diagnostic criteria such heightened impulsivity, emotional dysregulation and risk-taking behavior already point in that direction, as well as the prevalence of adverse experiences during childhood. In extension to this, LHT would predict that people with BPD may show signs of high stress responsivity (which may be a distinguishing feature from antisocial personality traits or disorder, where a more unemotional reactivity pattern is typical), a lack of trusting relationships, unstable romantic relationships, high number of short-term sexual relationships as well as increased vigilance toward partners’ faithfulness, early biological maturation, and poor investment in own offspring [33]. Moreover, symptom patterns were expected to differ between men and women, with male patients showing more externalizing features and females showing more internalizing behaviors [29]. Furthermore, comorbid conditions of BPD should feature among those syndromes associated with a ‘faster’ LHS, including ADHD, perhaps except the inattentive type of ADHD, BD, substance abuse and bulimia nervosa (BN) [25].

## TRAITS ASSOCIATED WITH BPD FOLLOWING A FAST LH STRATEGY

### Neuropsychology

One key feature of BPD concerns patients’ difficulties in regulating their emotions in appropriate ways, which may account for several symptoms including idealization and derogation of others, impulsivity and risk-taking behavior. These signs and symptoms can be conceptualized as behavioral expression of high stress responsivity. According to the Adaptive Calibration Model high stress responsivity promotes a fast LHS in dangerous and unpredictable contexts, whereby it increases vigilance to threat and down-regulates one’s sensitivity to social feedback [27, 34]. Consistent with this hypothesis, several studies have shown alterations of the hypothalamic–pituitary–adrenal stress axis in BPD, which correlate with symptom severity and a history of childhood trauma [35]. In fact, early adversity in general has been found to be associated with persistent changes of stress responsivity, possibly via epigenetic mechanisms [36]. Along similar lines, research into emotion perception suggests that patients with BPD display heightened vigilance or avoidance reactions to negative emotions such as fear and anger [37, 38]. At the same time, patients with BPD are often ‘alexithymic’, that is, they have difficulties in reflecting upon own and others’ emotions, whereby alexithymia in BPD has been found to be related to stress intolerance and impulsivity [39]. This apparent ‘empathy paradox’ however is plausible considering LHS emerging from early adversity [40]. Linehan has argued that patients with BPD may be hypersensitive to emotional cues that potentially signal rejection or abandonment [41]. Such biased emotion perception impacts on social interaction, if it interacts with difficulties in emotion regulation arising from overactivation of the attachment system [5]. Overactivation of the attachment system leads to a functional down-regulation of mentalizing abilities, partly, as a means of self-protection against continuing traumatization by an abusive caregiver [5]. Accordingly, hypersensitivity toward negative emotions may further contribute to distorted views of others, such that others are generally perceived as untrustworthy [42, 43]. In turn, seeing others as untrustworthy and uncooperative may enhance one’s own (unconscious) opportunistic attitude toward short-term exploitation of resources [44].

This view is also compatible with research showing enhanced impulsivity and delay discounting in patients with BPD. In fact, if one’s inner working model suggests poor resource availability in the future (compatible with a fast LHS), immediate resource acquisition is a logical consequence. In line with predictions, empirical evidence suggests that patients with BPD are poor in impulse control and in tolerating delay of gratification, that is they prefer immediate (lower) gains over (higher) future monetary gratification [45].

### Personality traits and interpersonal behavior

Research involving theories of temperament and personality development suggests that a fast LHS would be associated with high scores on novelty seeking, low scores on cooperativeness and harm avoidance, and low scores on agreeableness and conscientiousness, whereby high scores on the latter two dimensions were more characteristic of slow LHS [25, 31, 46]. In addition, the exploitation of others is typical of Machiavellian personality traits [47].

Consistent with this hypothesis, one study reported higher scores on novelty seeking and lower scores on cooperativeness in BPD patients compared with nonclinical and clinical controls [48]. In another study, BPD patient scored higher on Machiavellianism than controls [49]. These findings are consistent with the hypothesis of a fast LHS in BPD. Our own research group has utilized neuroeconomic games and responsivity of patients to the intranasal administration of a single dose of oxytocin (OT) to study LH-relevant behavior in BPD. For example, in a study using a Dictator Game version, in which participants had the option to punish observed unfairness occurring during an interaction of two characters, we found differences in personality traits between BPD patients and controls, which had diametrically opposite impact on participant’s motivation to engage in third-party punishment. In line with predictions regarding the association of personality traits with a fast LHS, patients with BPD scored higher than controls on Machiavellianism, and lower on agreeableness and conscientiousness. Most interestingly, in BPD third-party punishment correlated Machiavellianism (and with neuroticism), and inversely with agreeableness (as a measure of empathetic concern for others), which was the reverse in nonclinical controls. This finding is consistent with the interpretation that patients with BPD seemed to pathologically identify with the disadvantaged person in the Dictator Game, whereby antisocial traits motivated patients to punish unfair behavior, rather than empathic concern for others [50].

In a similar vein, research into interpersonal trust and cooperation has revealed that individuals with BPD have difficulties in maintaining and re-establishing reciprocal trusting relationships. For example, King-Casas *et al.* used a so-called trust game (TG), where one player (the investor) is endowed with a sum of money units (MUs), of which he or she can ‘invest’ a proportion of his choice in another player (the trustee) [51]. The trustee then decides how much he or she is willing to return to the investor (as a measure of reciprocality and cooperation). Mistrustful investors are less likely to spend a substantial share, because they would expect an insignificant return by the trustee. Conversely, mistrustful trustees unlikely reciprocate, if the TG is played iteratively with the same investor, because they probably expect the investor to defect over time. BPD patients, as trustees, initially returned as many MUs as controls. However, contrary to controls, patients’ willingness to reciprocate diminished over successive rounds. Moreover, when the investor’s behavior was experimentally manipulated such that the trustee was frustrated by the lack of the other player’s cooperation, psychologically healthy subjects could be coaxed back into cooperation by overly generous investments, whereas BPD patients did not respond to cajoling [51]. In further support of a fast LHS associated with BPD, Unoka *et al.* found that BPD subjects, in the role of an investor in a TG, transferred fewer MUs than patients with depression and healthy controls, depending on symptom severity such as stress-related paranoia and difficulties in interpersonal relations, as well as with a lack of confidence in the trustee (i.e. reduced trust) [52]. Likewise, another study reported that patients with BPD, as investors, adjusted their investment in that they transferred fewer MU to unfair trustees while ignoring—unlike nonclinical controls—the trustee’s neutral or negative facial expression [53]. These findings are therefore compatible with the view that BPD patients act in quite opportunistic ways and disregard emotional signals of others that might guide one’s decision of whether or not to cooperate with others.

Another feature, often considered pathognomonic for BPD, is self-injurious behavior. Self-harm may occur in BPD in situations in which patients feel detached from their social environment or have activated their attachment system in the fear of being abandoned. While self-injury can be seen as the expression of the inability to differentiate inner experience from reality, an evolutionary view suggests that self-harm can also be a strong signal addressed at perceived attachment figures, including therapists [5]. In humans, parental care for offspring is extremely expanded, such that a threat posed by offspring to terminate one’s life is a menace to the biological fitness of the parents themselves. Put another way, self-imposed threat to the physical existence by offspring is perhaps the strongest signal on the side of the offspring to increase parental care and nurturance, and this may well be transferred to therapeutic relationships [54].

### Sexuality and mating

According to Del Giudice *et al.*’s Adaptive Calibration Model, a fast LHS would predictably be associated with increased risk-taking, earlier sexual intercourse and larger numbers of sexual partners. In addition, biological maturation is expected to be accelerated [24, 27, 29]. Indeed, a large population-based study revealed that early age at first sexual intercourse predicted lifetime number of sexual partners and future risk-taking behavior in general [55]. With regard to BPD, several studies have found that women with BPD engage earlier in sexual intercourse and have more sexual partners than nonborderline women [33, 56, 57]. In addition, BPD women experience more often partner violence, date rape and sexual coercion [56]. Moreover, comorbid substance abuse puts BPD subjects at risk for unprotected casual sex, sexually transmitted diseases and commercial sex work [58, 59]. Symptom severity of BPD is furthermore associated with teenage pregnancy, unplanned pregnancies and live births, but not number of abortions [60]. According to a recent survey in over 100 in-patients with BPD, a majority reported significantly more sexual partners in the past 12 months than healthy controls, BPD subjects also expected to have more sexual partners in the near future than controls, and they reported a greater willingness to engage in risky health behaviors, but not financial risks (Brüne M, Edel M-A, Decker C, Schojai M., unpublished work). In further support of the idea that BPD reflects a fast LHS, individuals with BPD are more likely to experience breakups of relationships [61], even though individuals with borderline features engage more in costly mate retention tactics, whereby monopolization of time, emotional manipulation, commitment manipulation, violence against rivals, submission and debasement, and verbal possession signals are more frequently observed in men, whereas jealousy induction, derogation of competitors and derogation of the mate are more prevalent in women [62]. This is compatible with a fast LHS, because these mate retention tactics are more likely to work effectively in the short term, but less so in the long run. This may be so, because they are costly to the pursuer, and aversive to one’s mate, which may, in fact, increase the likelihood of a breakup [63].

In contrast to the idea that BPD reflects a fast LHS, there is no evidence so far for an earlier somatic maturation such as age at menarche in BPD [33, 57]. This poses a serious drawback on the theoretical conceptualization of BPD as a pathological variation of a fast LHS. Research in nonclinical youths suggests, however, that younger age at menarche in girls is associated with increased risk for psychopathology [64, 65]. For example, early maturing girls exhibit higher levels of internalizing stress and aggression, particularly those who have experienced emotional numbing in response to peer stress [66]. Precocious menarche also seems to nongenetically impact on the development of conduct disorder in girls [67]. Taken together, these studies suggest that earlier sexual maturation in girls is associated with sub-threshold BPD or at least with important ‘core’ features of BPD.

### Parenting

A fast LHS would not only be compatible with high mating effort, it would also be associated with low parental effort. In fact, invalidating parenting may be one mechanism involved in the transgenerational transmission of BPD personality traits [41]. In line with the hypothesis of a fast LHS in BPD, mothers with BPD seem to display critical and intrusive behaviors, as well as role confusion (i.e. fear of being abandoned by own offspring) and frightened or frightening behaviors. This oscillation between over-involvement and withdrawal as well as between hostility and coldness seems to be characteristic of mothers with BPD [68]. Our own observation in an in-patient sample of patient with BPD seems to corroborate this conclusion. We found that a relatively large number of patients with BPD came from a family background in which the biological father was absent, or multiple consecutive stepfathers had been present during childhood and adolescence of the affected individual. Moreover, several patients have half-siblings from relationships of their mothers with multiple partners. Likewise, we observed among in-patients with BPD that a substantial number of women have been forced to give their children into foster care or under the auspices of youth welfare services (Brüne *et al.*, unpublished work), which, from an evolutionary perspective makes sense in light of the assumption of a fast LHS.

## ARE THERE FEATURES OF BPD FOLLOWING A SLOW LHS?

Even though the overall pattern of behavior in BPD, as well as the underlying cognitive and emotional processes, implies a fast LHS, some traits associated with the syndrome are rather suggestive of a slow LHS. These could, in part, reflect compensatory mechanisms for behaviors at the fast end of the continuum. In fact, BPD is not a stable condition, and it could well be that ‘slowing’ (rather than ‘slow’) features emerge secondary to negative experiences following the pursuit of a fast LHS. As Del Giudice points out, while risky strategies may yield large gains in case of success, they also impose considerable costs in case of failure. For example, a defensive strategy in BPD could serve the purpose to avoid abandonment, which could explain why BPD patients score high on ‘harm avoidance’ [25, 46, 48]. However, as shown above, this does not seem to apply to sexual harm [55–60].

Another feature, typically found in individuals with BPD, is the tendency of patients to denigrate themselves, which may be expressed by feelings of emptiness or self-disgust. In fact, disgust seems to be a relevant factor involved in patients’ self-concepts, whereby the degree of disgust is often linked to the severity of traumatizing experiences [69]. High sensitivity to disgust interferes with a fast LHS, particularly in relation to sexual behavior. Conversely, insensitivity to disgust may bare the risk of contracting sexually transmitted diseases [25]. Following this line of reasoning, the presence of disgust could be an indicator of a slowing LHS, even though it seems relevant to distinguish between pathogen, moral and sexual disgust, whereby the latter two correlate with conscientiousness and agreeableness in nonclinical subjects, which is implausible in the case of BPD, because conscientiousness and agreeableness are usually low in BPD [70].

## NEUROIMAGING

Abundant evidence suggests that childhood maltreatment is associated with reductions in volume of limbic areas and the corpus callosum, and that impulsivity in BPD is associated with alterations in blood flow in frontal cortical regions [71–74]. While this review cannot summarize all relevant neuroimaging findings in BPD, an important issue with regard to the interpretation of neuroimaging data concerns the view suggesting that alterations in brain metabolism or structure do not necessarily reflect defective functioning. According to Teicher *et al.*, early environmental stress, e.g. in the form of childhood neglect or abuse, is possibly not simply toxic to the brain, thus interfering with (normal) brain development [73]. Instead, ‘exposure to significant stressors during a sensitive developmental period causes the brain to develop along a stress-responsive pathway’, thereby eliciting ‘a cascade of stress responses that organizes the brain to develop along a specific pathway selected to facilitate reproductive success and survival in a world of deprivation and strife’ [73]. This fundamentally different view of structural and functional brain imaging findings is in full accordance with the Adaptive Calibration Model according to which early experiences not only shape the psychological development of inner working models and how individuals adapt their LHS according to their predictions of future resource availability, but also that early experiences leave a mark on how the hardware (i.e. the brain) supports the operation of one’s individual software (i.e. inner working model) [27]. In the case of BPD, this suggests that alterations in limbic structure may actually support a fast LHS.

## GENETICS

A recent review concluded that despite evidence for heritability of around 40% of BPD, the search for candidate genes involved in BPD has been disappointing, which could relate to the ‘tendency to look for genetic effects on disease rather than genetic effects on vulnerability to environmental causes of disease’ [9]. Generally speaking, research into psychiatric genetics has largely focused on the diathesis-stress model, according to which subjects are vulnerable to develop a disorder if carrying a genetic variant that meets some sort of adversity or negative life event [75]. Conversely, some genetic variation may protect against the development of a disorder even in the presence of severe adversity [76]. The diathesis stress model can, however, not explain why so many ‘vulnerability genes’ have undergone recent positive selection in human evolution. This is contradictory in itself, because it is implausible to assume that natural selection has favored allelic variants, which increase vulnerability to adversity [77]. Instead, this strongly suggests that these genes exert hitherto undetected or overlooked beneficial effects with regard to reproductive fitness (which is not necessarily the same as ‘good for health’) [24]. Accordingly it has been argued that a particular genetic variation that predisposes to pathology if associated with early adversity can have beneficial effects when environmental contingencies are developmentally more supportive [78, 79]. This suggests that it is more accurate to speak of differential susceptibility or plasticity conferred by genetic variation—i.e. responsivity to both positive and negative conditions—rather than focusing one-sidedly on vulnerability, whereby plasticity genes can have additive effects, that is the susceptibility to the environment may increase with the number of plasticity alleles [80, 81]. It is therefore plausible to assume that the same genetic polymorphism can be linked to a ‘faster’ or ‘slower’ LHS, depending on the quality of early environments.

A look into genes involved in OT turnover may exemplify this view. Genes coding for the oxytocin receptor (OXTR), genes coding for OT and genes that indirectly contribute to OT expression such as CD38 have been linked to social cognition and interaction including quality of marital relationships, as well as childhood problems, which renders them interesting candidates for research in BPD [82–85]. Moreover, imaging genetic studies suggest that polymorphic variation of the OXTR gene is associated with structural and functional differences in limbic structures, which are known to contribute to emotion regulation, a key dysfunction in BPD [86].

Indirect evidence from studies in nonclinical samples linking the OXTR with childhood adversity, insecure attachment and emotion dysregulation indicate that the OXTR may also play a role in BPD or subthreshold phenotypes. From an LHT perspective, one would expect that one allele would convey plasticity, whereby the association with early adversity would more likely lead to a fast LHS, and association with supportive environments would produce a slow LHS. The phenotype associated with the other allele would be unresponsive to environmental influence. In partial support of this idea, gene–environment interaction between childhood maltreatment and both emotional dysregulation and attachment style that was moderated by polymorphic variation of the OXTR gene, whereby homozygous G-carriers of the single nucleotide polymorphism (SNP) rs53576 showed more pronounced emotional dysregulation and disorganized attachment patterns when exposed to childhood trauma compared with A/G or A/A allele carriers [87]. In contrast, parental emotional warmth and family stability compensated, in part, for the effects of traumatic experiences on mood and resilience in carriers of at least one G allele [88]. Along similar lines, individuals who experienced childhood maltreatment were susceptible to developing depression when carrying at least one G allele, whereas A/A carriers were less responsive to early adversity [89].

Conversely, a recent study reported a diametrically opposite finding, whereby A-allele carriers of the same SNP had high levels of BPD symptoms when raised by depressed mothers and low levels when grown up in families with nondepressed mothers. GG homozygotes were unresponsive to early rearing conditions, suggesting that the SNP rs53576 of the OXTR gene could confer ‘differential susceptibility’ to environmental contingencies [90]. In keeping with differential susceptibility models, another study reported that girls were at greater risk of developing BPD symptoms when carrying at least on A allele of the SNP rs53576 and when experiencing childhood maltreatment, whereas maltreated boys were more vulnerable to developing BPD symptoms when being homozygous for the G/G allele [91]. The opposite genotypes were unresponsive to family environment in both sexes. Notably, among boys the G/G carriers were less likely to show BPD symptoms when growing up in nonmaltreating family environments with no comparable effect in female A/A carriers, which led the authors to suggest that differential susceptibility occurs solely in boys [91].

In summary, these findings, though in part contradictory, suggest that variation of the OXTR gene is involved in individual differences in susceptibility to adversity and hence, the development of BPD symptoms. However, OT is certainly not the only, and most likely not the most important neuromodulator involved in the regulation of stress responsivity and LHS. In any event, it may nevertheless be helpful to consider the view that genetic polymorphisms involved in a psychiatric condition may not simply confer vulnerability, but possibly act in protective ways depending on early environment [24, 92]. As regards BPD, it is currently unclear whether individuals being at risk of developing the condition carry a larger than average number of plasticity alleles, which in combination with early adversity produce a BPD phenotype. Future genetic studies should address this question more explicitly.

## COMORBIDITY

The spectrum of comorbid disorders associated with BPD is mixed, with ADHD being suggestive of a fast LHS, whereas the case for PTSD and depression is more complex. Studies suggest that comorbidity rates of these disorders with BPD are considerable [93, 94]. ADHD is associated with increased impulsivity, novelty-seeking and other externalizing features indicative of a fast LHS [95]. PTSD seems to feature the extremes of variation of defense mechanisms akin to arrested flight, submission, freezing and dissociation [96, 97]. Both PTSD and depression can be situated at both ends of the fast-slow LHS spectrum. As for the fast end, hypervigilance and highly reactive stress regulating mechanisms can have adaptive properties in dangerous environments (i.e. promoting a fast LHS), yet they may also bare the risk of dysfunction. Accordingly, PTSD and depression could be a costly consequence of a failure of stress regulation. Consistent with this interpretation, depression is more likely to occur in fast maturers, somatic symptoms associated with depression are linked with early adversity and depression in adolescence often co-occurs with externalizing behaviors, and generally with lower agreeableness, conscientiousness and poor inhibitory control [25]. Along similar lines, Belsky *et al.* have argued that internalizing problems—which are typical for many women with BPD—may ultimately serve to lower metabolism, increase body fat and thus initiate menarche earlier [29]. It is equally plausible, however, to assign low mood a role in slow LHS, because it may shield an individual from pursuing unattainable goals and help avoid risks. With regard to BPD, either explanation may apply, that is depression could be the cost for failure of a high-risk (fast) strategy, or a self-protective mechanism in the sense of down-regulating strategic action to cope with stress caused by a fast LH pattern.

Along similar lines, eating disorders may reside at both ends of the continuum of LHS, based on the relevance of sexual competition for mates. Accordingly, a slow LHS would promote females to desire a thinner body than what men perceive sexually most attractive, which in turn, would increase the woman’s value as a long-term mate [25]. Consequently, slow LHS should be more characteristic of anorexia nervosa (AN) than BN [98]. Consistent with this hypothesis, BN is associated with earlier sexual maturation and activity; patients with BN also show more externalizing behaviors than patients with AN. In accordance, BPD seems to be more often associated with BN than AN [99]. However, more evenly distributed comorbidity rates have been reported in other studies, e.g. [100].

## DISCUSSION

Seen through the lens of Behavioral Ecology, there is abundant evidence in support of the idea that BPD reflects a pathological variant of a fast LHS [33]. In addition, insights from research into the neuropsychology, personality traits, interpersonal behavior, neuroimaging findings and genetics of BPD corroborate this view. While there is still controversy over differences in prevalence rates of BPD in men and women, there is overwhelming evidence for the prediction from LHT suggesting that men show more aggressive and noncompliant behavior (akin to antisocial personality traits), whereas women more often show signs of internalizing behavior, including signs of depression and anxiety [101, 102]. Accordingly, male BPD is more often characterized by explosive temperamental features and higher levels of novelty seeking compared with female BPD [103]. With regard to personality, antisocial traits or full-blown antisocial personality disorder is more common among men with BPD. In addition, men with BPD have more often than women comorbid substance use disorders. By comparison, women with BPD are more often diagnosed with comorbid eating disorders, depression and anxiety, and PTSDs, all of which is consistent with predictions from LHT [11, 104].

Why is all this interesting in regard of public health issues? First, the behavioral ecological view on BPD may have important ramifications for the psychiatric treatment of this condition. For one, neuroimaging studies of BPD may be worth reconsidering. In contrast to the traditional ‘medical’ perspective suggesting that deviations from a statistical norm represent ‘deficits’ (i.e. brain damage), neuroimaging findings in BPD may, in fact, reflect complex adaptations to early adversity and thus serve stress-regulation purposes, which may be functional in dangerous and unpredictable environments, but dysfunctional in safer environments [73]. So, in keeping with the Adaptive Calibration Model, a therapeutic stance could entail acknowledging that a patient’s personal history has impacted on his or her stress regulating mechanisms which include brain circuits involved in threat evaluation and prediction of future resource availability [105]. This attitude is fundamentally different to a more fatalistic ‘brain damage’ perspective. Of note, studies have shown that anatomical ‘abnormalities’ found in patients with a history of childhood adversity are reversible upon psychotherapy, suggesting that functional or structural brain variation is not necessarily impervious to modification [106].

Along similar lines, LHT suggests that the one-sided view on psychiatric genetics (vulnerability concept) should, in part, be replaced by one that considers genetic variations as expression of plasticity ‘for better or worse’, depending on the interaction of genes with the environment [81, 107]. This is a crucial point, because the same allelic variation can promote a slow or a fast LHS, depending on early environmental contingencies, thus acting at both ends of the LHS spectrum [27]. This view may have profound implications for the understanding of BPD, because BPD patients may actually be among the genetically most plastic individuals who, due to early adversity, have developed dysfunctional interpersonal strategies [108].

Another example for how interpretation can influence therapeutic perspectives comes from studies in BPD using neuroeconomic paradigms. Commenting on King-Casas *et al.*’s TG study, Kishida *et al.* noted ‘borderline personality disorder confers a ‘diminished capacity’ to represent expectations for social partners, and as a consequence individuals with BPD ‘cannot take corrective action’ (social control signal) that might serve to re-establish cooperative interaction’ (this author’s italics) [51, 109]. An alternative interpretation of the same finding that is in line with LHT suggests that, rather than reflecting a cognitive deficit, it is the motivational structure of patients with BPD that lead them not to take corrective action by reinstalling cooperation. That is, individuals whose inner working models suggest that others are untrustworthy may not be ‘motivated’ to respond to attempts to entice them back into a cooperative relationship [44].

As regards psychotherapy in general, existing treatments for BPD patients that have proved to be effective—dialectic behavioral therapy, transference-focused therapy, mentalization-based treatment, as well as newer developments including metacognitive interpersonal therapy and compassion-focused therapy (CFT)—have barely taken into account evolutionary aspects, with the exception of CFT [110, 111]. However, potential implications from LHT have entirely been disregarded so far. This review contends that it could help patients change interpersonal attitudes and expectations, as well as their ‘real-life’ behavior, if they gained insight into the inappropriateness of their current behavior considering present-day environmental conditions. Put another way, a ‘fast and furious’ LHS may make sense in unpredictable and dangerous conditions, but less so in relatively safe and reliable circumstances. Of course, this cannot simply be ‘taught’, but worked-through over time in insight-oriented psychotherapeutic approaches [53]. As Fonagy put it, ‘we are likely to see behavioral organizations that we currently term personality disorders as age-specific adaptations to biopsychosocial pressures, which are best treated by developmentally specific interventions’ [112].

The behavioral ecological approach has several limitations in explanatory power. One is that BPD is a fairly heterogeneous syndrome. Given that five out of nine diagnostic criteria are necessary for a diagnosis, it also follows that two randomly picked patients with BPD may overlap in only one symptom [113]. Accordingly, the LH model presented here may not fit all phenotypic variations of BPD. (An alternative would be to develop a novel taxonomy of psychiatric disorders solely based on predictions from LHT, but such a re-launch ‘from scratch’ would disregard that diagnostic systems such as the Diagnostic and Statistical Manual of Mental Disorders, 5th edition (DSM-5) have evolved just like ‘memes’, and so has medical education. Thus, it would put evolutionary approaches to psychiatric conditions at risk of not being seriously considered by clinicians at all.) Moreover, human behavior is extraordinarily malleable and plastic, such that signs and symptoms change over time. In the case of BPD, features most indicative of a fast LHS such as risk-taking behavior, impulsivity and self-mutilation decline in severity with increasing age [114]. This is also predictable from LHT, because LHS that aim at maximizing reproductive success early in life become less relevant with increasing age, and should be negligibly present beyond the reproductive lifespan, i.e. in post-menopausal women.

From an LH perspective, future research should aim at collecting quantitative data about survival, reproduction and gene replication in large clinical samples. These data should include a detailed description of the various behavioral phenotypes according to LHT criteria (beyond DSM diagnoses) and individuals’ early and current environmental conditions.

Although no such data exist to date, a large study of fecundity in different psychiatric disorders found that those conditions that qualify best as ‘fast’ LHS [95], including BD, substance abuse (and in part, depression) are not associated with reduced fecundity (or even better than average fecundity), while those that may follow a ‘slow’ LHS (e.g. autism) are associated with reduced fecundity [115]. To answer the question whether or not ‘subthreshold’ or ‘diluted’ phenotypes are associated with reproductive advantages or disadvantages, there is a need for epidemiological studies in large nonclinical samples that are well characterized according to character and personality dimensions.

Related to this, a final point of interest for public health concerns how psychiatric diagnoses are made. An LH perspective suggests that the decision over ‘disorder’ versus ‘no disorder’ is not a matter of unconditional veracity, but highly dependent on contextual including cultural factors [95]. Potential implications for psychiatric nosology cannot be exhaustively discussed here, however, as the case of BPD may illustrate, the functional analysis of ‘problem behavior’ in an evolutionary perspective may come to different conclusions compared with views from social science perspectives [29]. An LH approach to psychiatric conditions does not imply that disorders are, in general, adaptive. On the contrary, it is explicitly contended that BPD is not an adaptive condition. However, sub-threshold phenotypical trait expression may be adaptive in specific (here, unpredictable) circumstances, especially when considering that ‘adaptive’ in a biological sense does not entail well-being or physical and mental health [116].

‘Dis-order’ arises from the inappropriateness of cognitions, emotions and behavior in a given environmental context. This can leave long-lasting or even permanent marks on the central nervous system and the way interpersonal processes are ‘embodied’. Psychiatry needs to take on the challenge to not emphasize boundaries between ‘disease’ and ‘normalcy’, particularly in light of waxing and waning weights assigned to the ‘bio’, the ‘psycho’ and the ‘social’ aspects of psychiatric conditions, whereby evolutionary approaches may be helpful to integrate these aspects into a more coherent framework for psychiatric conditions.
